# Decoding the Kruppel-like Transcription Factors in Atherosclerosis: Insight from Molecular and Translational Perspectives

**DOI:** 10.3390/ijms262311714

**Published:** 2025-12-03

**Authors:** Yiyang Cao, Chenyue Wang, Xuzhou Zhu, Meiqian Zhan, Jinhui Xu, Meixiu Jiang

**Affiliations:** 1 The Queen Mary School, Jiangxi Medical College, Nanchang University, 999 Xuefu Road, Nanchang 330031, China; 4217123202@email.ncu.edu.cn (Y.C.); chenyuew@email.ncu.edu.cn (C.W.); 4217123192@email.ncu.edu.cn (X.Z.); 4203122371@email.ncu.edu.cn (M.Z.); 4217123195@email.ncu.edu.cn (J.X.); 2 Jiangxi Province Key Laboratory of Bioengineering Drugs, The National Engineering Research Center for Bioengineering Drugs and the Technologies, Institute of Translational Medicine, Jiangxi Medical College, Nanchang University, 999 Xuefu Road, Nanchang 330031, China

**Keywords:** Kruppel-like transcription factors, atherosclerosis, clinical treatment

## Abstract

Atherosclerosis is a leading contributor to cerebrovascular and cardiovascular diseases, which can be driven by multiple pathological processes, including chronic inflammation, lipid dysregulation, and vascular remodeling. Currently, lifestyle intervention and pharmacological intervention, like statins, are recommended in clinical treatments. However, the mortality and morbidity rates caused by atherosclerosis remain high. Kruppel-like transcription factors (KLFs) are zinc-finger-containing transcription factors that are involved in various physiological and pathological processes. By modulating endothelial cell homeostasis, smooth muscle cell phenotypic switching, and inflammatory responses, members of the KLF family—particularly KLF2, KLF4, KLF5, KLF6, and KLF14—emerge as pivotal regulators in the initiation and progression of atherosclerotic lesions. In this review, we constructed a comprehensive network of KLFs in the pathogenesis of atherosclerosis. Based on the molecular mechanism, this review for the first time highlighted newly identified substances that exploit KLF-modulated pathways to attenuate atherosclerosis, and discussed emerging gene therapy and nanotechnology approaches, addressing both the therapeutic promise and challenges associated with targeted KLF modulation. This first offered new avenues for translational and precision medicine in atherosclerotic cardiovascular disease from the perspective of KLF.

## 1. Introduction

In recent years, atherosclerosis has emerged as a significant global health concern. There has been a consistent rise in the global incidence of all three clinical manifestations of atherosclerosis: ischemic heart disease, ischemic stroke, and peripheral arterial disease, from 1990 to 2019 [1]. With the increasing risk posed by atherosclerosis, two problems should be considered: a substantial proportion of individuals with peripheral artery disease remain undiagnosed, and those diagnosed may not receive adequate prevention or treatment [2]. The lack of precise biomarkers for early diagnosis of atherosclerosis highlights the urgent need to explore new therapeutic targets and more efficacious drugs [3].

Atherosclerosis is a common cardiovascular condition marked by persistent inflammation, lipid accumulation, immune cell infiltration, and fibrous plaque formation within the arterial walls. It can lead to clinical manifestations such as angina, myocardial infarction, transient ischemic attacks, and peripheral artery disease, which result from plaque-induced constriction of blood vessels and reduced blood flow, thus increasing the risk of ischemic damage to vital organs such as the heart and brain [4]. The underlying mechanism begins with endothelial cell dysfunction, which is exacerbated by risk factors such as hyperlipidemia, hypertension, smoking, and inflammation. These conditions enhance arterial permeability to low-density lipoproteins (LDL), whose oxidative modification induces the expression of endothelial adhesion molecules through NF-κB signaling, facilitating the attachment and migration of monocytes and T-lymphocytes. Monocytes differentiate into macrophages that excessively take up oxidized LDL and transform into foam cells [5], a crucial component of an atherosclerosis lesion, while T-lymphocytes secrete cytokines like interleukin (IL)-1β and tumor necrosis factor (TNF)-α through NOD-like receptor protein 3 (NLRP3) inflammasome activation to amplify the inflammation [4]. Subsequently, inflammation-related cytokines and growth factors drive vascular smooth muscle cells (VSMC) from the media to the intima, where they proliferate and produce extracellular matrix components, thickening the fibrous cap through the tumor growth factor (TGF-β)/Smad signaling pathways [6]. As the plaques grow, they become unstable and may rupture, leading to blood coagulation at the rupture site and further occluding downstream veins or arteries [4,5,7].

Nowadays, the Kruppel-like factor (KLF) family has attracted significant attention due to its extensive functions in vascular biology [8]. KLFs are zinc-finger-containing transcription factors that regulate the transcription of target genes by specifically recognizing and binding to the GC-rich proximal promoter regions with CACCC elements in DNA via three C2H2-type zinc fingers [9,10,11]. To date, researchers have identified 18 members of the KLF family in mammals, which are diverse in tissue distribution, expression patterns, and functions. The KLF family plays a crucial role in numerous biological processes, including cell growth, differentiation, programmed cell death, inflammatory reactions, and metabolic control [12]. Accumulating evidence has demonstrated that specific KLF proteins exert essential regulatory effects on cellular functions within vascular contexts, particularly in vascular endothelial cells and smooth muscle cells. This dual involvement in fundamental biological processes and vascular cell regulation enables the KLF family to modulate key atherogenic processes like inflammatory response, thrombosis, vascular remodeling, and lipid metabolism [8], and it is therefore closely associated with atherosclerosis. Notably, the abnormal expression of KLF family proteins has been linked to a range of health issues, including various cancers, thrombus formation, diabetes mellitus, neurodegenerative diseases, and atherosclerosis [8,13,14,15,16]. For instance, the downregulation of KLF2 promotes endothelial inflammasome activation and contributes to atherosclerotic lesion formation [17], while KLF4 inhibits the dedifferentiation of VSMCs from a contractile to a mesenchymal-like phenotype, and is associated with several pro-inflammatory genes that promote the development of atherosclerosis [18]. Consequently, the investigation of KLFs as molecular markers and prospective therapeutic targets may serve as a crucial foundation for future atherosclerosis treatment and prognosis.

Accordingly, this review is grounded in the research conducted within this domain to elucidate the role of KLFs in atherosclerosis, including mechanistic and clinical treatments, and to obtain a comprehensive overview of the advancements in research within this field of study.

## 2. KLF Family Members

So far, a total of 18 members of the KLF family have been identified in mammals. Based on phylogenetic and structural architecture, KLFs are divided into three different subfamilies consisting of: (1) KLF1, KLF2, KLF4, KLF5, KLF6, and KLF7 group, it binds to histone acetyltransferases (HAT); (2) KLF3, KLF8, and KLF12 group, it binds to C-terminal binding protein (CtBP); (3) KLF9, KLF10, KLF11, KLF13, KLF14, and KLF16 group, it binds to SIN3 transcription regulator family member A (Sin3A). KLF15, KLF17, and KLF18 have not been categorized in any of these groups because they contain no defined protein interaction motifs. The KLF family is structurally conserved in that they all have a DNA-binding region at the carboxyl-terminal (C-terminal) end and a transcriptional regulatory region at the amino-terminal (N-terminal) end. The DNA-binding region is highly conserved and includes the essential zinc finger domain for DNA interaction, while the amino-terminal region varies in sequence and contains transcriptional regulatory domains that facilitate protein interactions and regulate the genetic expression [8,19].

Zinc finger domains are common motifs in transcription factors. The most frequently encountered zinc finger motif is the C2H2 type, in which a zinc atom is tetrahedrally coordinated by two conserved cysteine and histidine residues that allow the domain to fold into a ββα structure. All KLF family members contain three conserved C2H2 zinc finger structures at the C-terminus, and each zinc finger structure has a fixed length: zinc finger structures 1 and 2 each consist of 25 amino acid residues, while zinc finger structure 3 contains 23 amino acid residues.

While the conserved C-terminal zinc finger domains ensure DNA-binding specificity, the variable N-terminal regions, which can be classified into transactivation domain (TAD) and transcriptional repression domain (TRD), largely determine whether KLFs function as transcriptional activators or repressors through recruitment of co-regulatory complexes [19]. In terms of activation mechanisms, some KLFs can bind to co-activators, called HATs. For instance, KLF4 can synergize with HAT to inhibit cell proliferation [9]. Conversely, for repression mechanisms, the PXDLS motif in the terminus of KLF3, KLF8, and KLF12 can bind to the CtBP co-inhibitory complex to inhibit transcription [8]. Another typical mechanism of transcriptional repression is mediated by the N-terminus Sin3-interacting domain, which recruits the mSin3A histone deacetylase complex [11].

**Figure 1:**
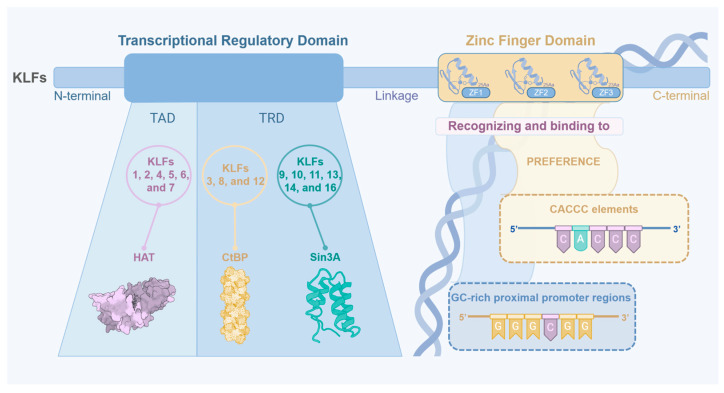
The structure and classification of KLFs. KLFs mainly consist of two parts: the zinc finger domain and transcriptional regulatory domains. The Zinc Finger Domain at the C-terminal recognizes and binds to CACCC elements and GC-rich proximal promoter regions (prefers to bind with CACCC elements). The transcriptional regulatory domains at N-terminal can be divided into transactivation domain (TAD) and transcriptional repression domain (TRD), which bind to specific molecules to perform different functions: For KLFs 1, 2, 4, 5, 6 and 7, it binds to histone acetyltransferases (HAT); For KLFs 3, 8 and 12, it binds to C-terminal binding protein (CtBP); For KLFs 9, 10, 11, 13, 14 and 16, it binds to SIN3 transcription regulator family member A (Sin3A).

Overall, these structural diversities (Figure 1) underpin the various physiological roles of KLF family members, particularly in the context of cardiovascular and metabolic diseases. This suggests that research in this field may provide potential value for the future treatment of atherosclerosis.

## 3. KLF-Mediated Regulatory Network in Atherosclerosis Pathogenesis

### 3.1. Regulatory Pathways of KLF2 in Atherosclerosis

As a member of the KLF family that controls atherosclerosis-related processes, KLF2 demonstrates endothelial protection and anti-inflammatory activity. Current studies researching KLF2 and atherosclerosis mainly focus on two aspects: blood flow and inflammation.

Physiological laminar flow (steady flow) maintains endothelial cell (EC) homeostasis, whereas pathological flow patterns, including turbulence, vortices, and low wall shear stress, promote vascular pathologies such as atherosclerosis and cerebral aneurysm formation [20]. S-flow activates KLF2 and KLF4, transcription factors critical for suppressing atherosclerosis by modulating endothelial nitric oxide synthase (eNOS)-associated pathways, including cGMP-PKG, cAMP, and insulin signaling. Consequently, KLF2 enhances nitric oxide (NO) bioavailability, improving endothelial-dependent vasodilation and inhibiting atherogenesis [21]. The shear stress sensor heart-of-glass 1 activates the MEKK3-MEK5-ERK5-MEF2 axis via interaction with Krav interaction trapped 1, inducing KLF2 and KLF4 expression to protect ECs [22,23]. Mechanistically, unidirectional shear stress (USS) and oscillatory shear stress (OSS) exert opposing effects: USS shows protective effects by upregulating KLF2, whereas OSS promotes pyroptosis-associated inflammasomes by inhibiting KLF2 expression, aggravating endothelial inflammation and plaque formation [17]. Additionally, OSS can activate the coxsackievirus-adenovirus receptor (CAR), which exacerbates atherosclerosis by promoting neutrophil trans-endothelial migration. However, this pro-atherogenic process is suppressed by the KLF2/activator protein-1 (AP-1) axis [24]. These findings further highlight the critical interaction between OSS and KLF2 in the pathogenesis of atherosclerosis.

**Figure 2:**
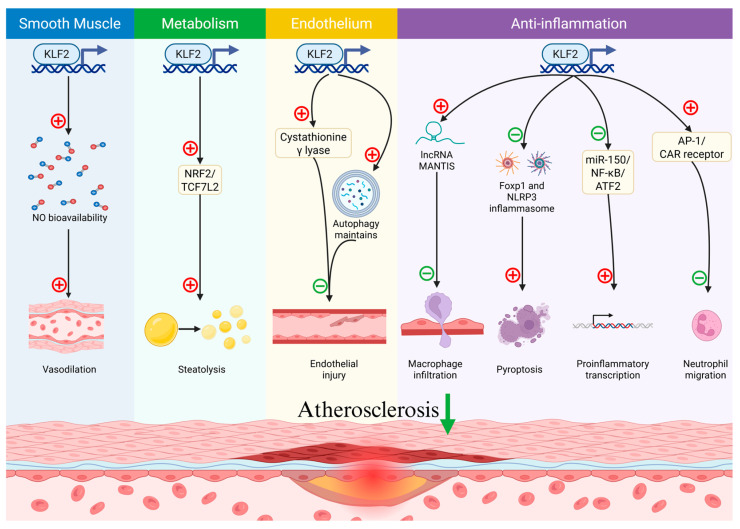
The schematic illustration of the beneficial roles of Kruppel-like transcription factor 2 (KLF2) in atherosclerosis. KLF2 has multiple beneficial roles in atherosclerosis. For vascular smooth muscle, KLF2 enhances nitric oxide bioavailability, thereby promoting vasodilatory responses. For metabolism, KLF2 promotes steatolysis through nuclear factor erythroid 2–related factor 2 (NRF2)/Transcription factor 7-like 2 (TCF7L2) pathway; For endothelium aspect, KLF2 prevents endothelial injury through activation of cystathionine γ-lyase and maintaining autophagic function; For anti-inflammatory effects, KLF2 inhibits proinflammatory responses through multiple mechanisms: reducing macrophage infiltration via lncRNA MANTIS, suppressing the forkhead box protein p1 (Foxp1) and NOD-like receptor protein 3 (NLRP3) inflammasome to inhibit pyroptosis, repressing proinflammatory transcription by silencing the miR-150 promoter and inhibiting NF-κB and activating transcription factor 2 (ATF2), and blocking coxsackievirus-adenovirus receptor (CAR)-mediated neutrophil migration via the KLF2/activator protein-1 (AP-1) axis. In the diagram, red “+” symbols (promotion) and green “−“ symbols (inhibition) are used to indicate how KLF2 influences various biological processes involved in atherosclerosis.

Moreover, the development of atherosclerosis is aided by endothelial dysfunction brought on by oxidized low-density lipoprotein (ox-LDL), which is typically an inflammatory reaction. Recent studies have ascertained multifaceted protection effects of KLF2 in this process through: (1) Inhibiting NF-κB to mitigate ox-LDL-induced endothelial inflammation [25], or upregulating VSMC-derived lipoprotein lipase (LPL) expression by including nuclear factor erythroid 2–related factor 2 (NRF2), which facilitates Transcription factor 7-like 2 (TCF7L2) expression, a transcription factor activating LDL promotor [26]. (2) Repressing constitutive proinflammatory transcription by activating transcription factor 2 (ATF2, a transcription factor inducing plaque formation) inhibition [27]. (3) Restoring peroxiredoxin 6 sulfhydration via miR-27b-dependent cystathionine γ-lyase activation, counteracting IL-1β-driven oxidative stress [28], or KLF2 also attenuating inflammatory response via other inflammation-related miRNA regulation, like miR-150, by silencing its promotor [29]. (4) Attenuating ICAM-1 induced monocyte (especially the macrophage) recruitment via long non-coding RNA (lncRNA) MANTIS motivation [30,31]. (5) Stimulating brain-derived neurotrophic factor-hexokinase 1 signaling to suppress NLRP3 inflammasome formation and endothelial pyroptosis [32]. Notably, beyond pyroptosis, KLF2 maintains autophagy function (another type of programmed cell death (PCD)) by suppressing MLKL expression [33]. In the condition of hypertension, KLF2 deficiency disrupts autophagy, accelerating atherosclerosis [34]. These findings summarize the protective effects of KLF2 (Figure 2) on atherosclerosis and highlight KLF2 as a therapeutic target, warranting exploration of KLF2 agonists and pathway-specific modulators for atherosclerosis treatment.

### 3.2. Regulatory Pathways of KLF4 in Atherosclerosis

Genome-wide association studies link 39% of human coronary artery disease (CAD) loci to genes associated with KLF4 binding sites, highlighting the regulatory significance of KLF4 in CAD pathogenesis [35]. Similar to KLF2, KLF4 exerts protective effects in atherosclerosis through EC protection, inflammation modulation, and PCD regulation.

Although KLF4 and KLF2 act synergistically, their mechanistic pathways differ. KLF4 responds to both USS and OSS, attenuating atherosclerosis by suppressing TGF-β activity, which is mediated by the MEKK3/MEK5/ERK5/MEF2 cascade activation [22,36]. Additionally, KLF4 mitigates inflammation by regulating macrophage function, suppressing pro-inflammatory cytokine expression, and reducing monocyte-EC adhesion [30,37]. KLF4 also modulates PCD pathways: it counteracts oxidative stress, restores autophagy in hyperhomocysteinemia-induced VSMC dysfunction [34,38], inhibits ferroptosis by upregulating glutathione peroxidase 4 (GPX4) and solute carrier family 7 member 11 (SLC7A11) [39], and transcriptionally represses NLRP3 by binding to its promoter [40]. Collectively, these mechanisms—distinct yet complementary to those of KLF2—highlight the multifaceted protective role of KLF4 in atherosclerosis. Notably, KLF4 also promotes macrophage phenotypic change from pro-inflammatory M1 to anti-inflammatory M2 subtypes with two important pathways: transactivation of cholesterol 25-hydroxylase (Ch25h)/liver X receptor (LXR) regulatory axis via H3K4me3 and H3K27ac enrichment and demethylation, and inducing peroxisome proliferator-activated receptor gamma (PPARγ)/signal transducer and activator of transcription 6 (STAT6) signaling pathway by binding to the promoter of PPARγ, which eventually attenuates atherosclerosis [41].

**Figure 3:**
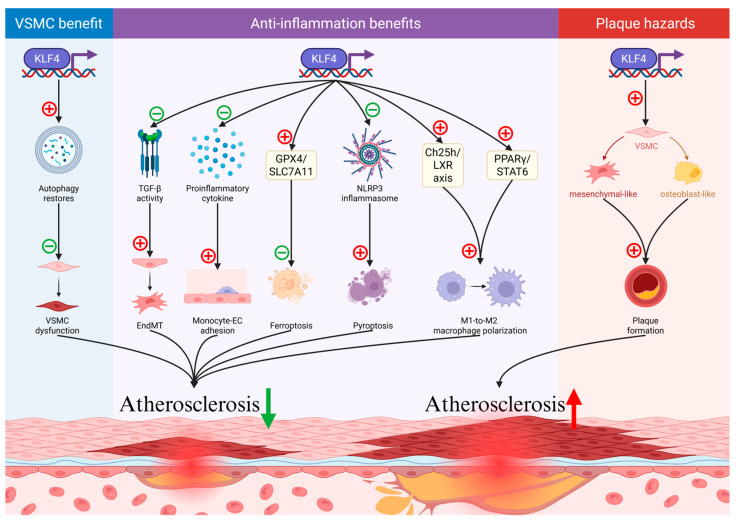
The schematic illustration of the controversial roles of Kruppel-like transcription factor 4 (KLF4) in atherosclerosis. KLF4 has both beneficial and hazardous roles in atherosclerosis. Regarding beneficial effects, KLF4 restores autophagy to prevent vascular smooth muscle cell (VSMC) dysfunction, suppresses endothelial-to-mesenchymal transition (EndMT) via suppressing transforming growth factor (TGF-β) activity, suppresses proinflammatory cytokines to inhibit monocyte-EC adhesion, and attenuates ferroptosis by upregulating glutathione peroxidase 4 (GPX4) and solute carrier family 7 member 11 (SLC7A11), and represses NOD-like receptor protein 3 (NLRP3) inflammasome, thereby mitigating pyroptosis. Additionally, KLF4 promotes M1-to-M2 macrophage polarization via the cholesterol 25-hydroxylase (Ch25h)/liver X receptor (LXR) axis and peroxisome proliferator-activated receptor gamma (PPARγ)/signal transducer and activator of transcription 6 (STAT6) pathway. Regarding hazards, KLF4 induces a switch of VSMCs from a contractile to a mesenchymal-like phenotype and drives SMC differentiation into Lgals3^+^ cells, which contribute to plaque formation. In the diagram, red “+” symbols (promotion) and green “−“ symbols (inhibition) are used to indicate how KLF2 influences various biological processes involved in atherosclerosis.

Nevertheless, the function of KLF4 is still controversial, due to its adverse effects on VSMC phenotypic change and plaque stability [42]. Research has identified that KLF4 activation in VSMCs drives a phenotypic shift from contractile to mesenchymal states, marked by CD34, CD44, and stem cell antigen-1/lymphocyte antigen 6 complex locus A expression [18]. Additionally, while Shou et al. demonstrated that miR-126 inhibits foam cell formation and promotes M2 polarization by downregulating KLF4 [43], KLF4 deletion in non-AdvSCA1-SMC enhances contractile phenotypes and protective macrophage recruitment, reducing necrotic core size and macrophage infiltration without altering overall plaque burden [44]. Alencar et al. further reported that KLF4 drives SMC differentiation into osteoblast-like Lgals3+ cells in advanced lesions, suggesting time-dependent roles [35]. These conflicting findings (Figure 3) underscore the need to clarify the spatiotemporal regulatory mechanisms of KLF4 and identify additional signaling pathways.

### 3.3. Regulatory Pathways of KLF5 in Atherosclerosis

Unlike KLF2 and KLF4, KLF5 aggravates atherosclerosis via non-coding RNAs networks, including miRNAs, lncRNAs, and circRNAs. For instance, miR-375 promotes endothelial angiogenesis in vitro by enhancing cell migration, proliferation, and vascular network formation. Mechanistically, miR-375 inhibits KLF5, which suppresses the KLF5/MerTK axis and KLF5/NF-κB pathway, thereby reducing inflammation and fostering angiogenesis [45,46]. Similarly, miRNAs such as miR-576, miR-145, and miR-10b-3p attenuate atherosclerosis by directly downregulating KLF5 [47,48].

In contrast to miRNAs, circRNAs, and lncRNAs indirectly modulate KLF5 by sequestering miRNAs. For example, Circ_0002984 binds let-7a-5p to upregulate KLF5, driving the proliferation and migration of ox-LDL-stimulated human vascular smooth muscle cells (HVSMC) [49]. As well, circUSP9X/miR-148b-3p/KLF5 and circ_USP36/miR-182-5p/KLF5 axes stimulate atherosclerosis similarly [50,51]. LncRNAs also contribute to KLF5 modulation. OIP5-AS1 protects ECs from ox-LDL damage by sponging miR-135a-5p to suppress KLF5 [52]. Conversely, LINC01123—elevated in serum and ox-LDL-treated VSMCs—promotes fibrous plaque progression by acting as a miR-1277-5p sponge to upregulate KLF5, accelerating VSMC migration and proliferation [53]. These findings highlight KLF5 suppression as a potential therapeutic strategy for atherosclerosis.

### 3.4. KLF6 Regulatory Pathways in Atherosclerosis

KLF6 has a biphasic effect on atherosclerosis. Protectively, KLF6 typically suppresses inflammatory factors and adhesion molecule production, reducing monocyte-endothelial adhesion and attenuating plaque progression [54]. KLF6 also regulates EC migration. During vascular injury, KLF6 translocates to the nucleus and enhances the transcriptional activity of TGF-β receptors, including endoglin and activin receptor-like kinase 1, which are beneficial for angiogenesis and vascular remodeling [55]. Research conducted by Cheng et al. further provided direct evidence that KLF6 promoted Ox-LDL-mediated angiogenesis [56].

Detrimentally, KLF6 contributes to disease progression via ncRNA-mediated pathways. Ma et al. demonstrated that miR-21a-5p downregulates KLF6 and ERK1/2 signaling, promoting M2 macrophage polarization and reducing macrophage infiltration [57]. Additionally, nicotine, a known atherogenic risk factor, upregulates LINC01272, thereby derepressing KLF6 and triggering macrophage pyroptosis [58]. These findings demonstrate that KLF6 might exacerbate atherosclerosis through context-dependent mechanisms, and further investigation into the spatiotemporal regulation of KLF6 is warranted to evaluate its potential as a therapeutic target.

### 3.5. KLF14 Regulatory Pathways in Atherosclerosis

Atherosclerosis progression can be driven by impaired cholesterol efflux, lipid accumulation, and chronic inflammation, while KLF14 arises as a critical regulator of cholesterol metabolism, enhancing the expression of the cholesterol efflux transporter, ATP-binding cassette transporter A1 (ABCA1), to attenuate atherosclerosis. This is corroborated by reduced ABCA1 mRNA levels in KLF14-deficient murine models [59]. Additionally, KLF14 downregulates LPL, an enzyme that hydrolyzes lipids to release fatty acids and glycerol, via a miR-27a-dependent pathway [60]. KLF14 further exerts anti-atherogenic effects by suppressing macrophage inflammatory responses through inhibition of MAPK cascades and NF-κB/p65 signaling, thereby reducing monocyte adhesion [61,62]. In summary, KLF14 exerts multi-target protective effects in atherosclerosis, while its antagonistic effects with family members such as KLF5 reveal the complexity and therapeutic potential of the KLF network regulation.

### 3.6. Regulatory Pathways of Other KLF Families in Atherosclerosis

Emerging evidence highlights the critical role of KLF family members in atherosclerosis pathogenesis, which promotes further investigation of the involvement of other KLF members in this disease. For example, KLF7 activate histone deacetylase 4 and also inhibit atherosclerotic-associated ferroptosis by transcriptionally activating AlkB homolog 5, thereby improving atherosclerosis [63,64]; KLF10 inhibit the expression of heme oxygenase-1, exacerbating oxidative stress and atherosclerosis, while controversially KLF10 deficiency promote the generation of pro-inflammatory macrophages, thus aggravating atherosclerosis [65,66]; KLF11 significantly inhibits the activation of inflammatory responses in ECs, serving as a novel protector against diabetic atherosclerosis [67]; KLF13 directly binds to the promoter of smooth muscle protein 22-alpha, thereby facilitating the phenotypic dedifferentiation of VSMCs and serving as a potential target for atherosclerosis treatment [68]; KLF15 attenuates atherosclerosis by suppressing vascular inflammation and regulating the phenotype of VSMC and macrophage [69,70,71,72]; KLF16 promotes the PPARα signaling pathway and inhibits the development of atherosclerosis [73]. They collectively present the significant role of KLF in atherosclerosis. However, conspicuously, more evidence and studies are required to analyze whether they are essential in atherosclerosis progression and valuable to further research.

## 4. Clinical Perspectives

### 4.1. Drugs Currently Used in Clinical Treatment Through KLF

A wide range of specialized medicines with clearly defined mechanisms of action and proven efficacy has been developed and is now widely used in the clinical management of atherosclerosis.

Statins lower LDL concentrations by inhibiting the rate-limiting enzyme in cholesterol biosynthesis, HMG-CoA reductase, thereby attenuating atherosclerosis and are widely recommended as first-line treatment for diagnosed atherosclerosis [74,75]. Notably, the combination of statin and ezetimibe, a kind of cholesterol absorption inhibitor, has shown synergistic effects in reducing cardiovascular risks [74,75,76]. PCSK9 inhibitors prevent LDLR degradation in hepatocytes, enhancing LDL-C clearance, which benefits patients who are intolerant to statins [76]. These therapies are clinically applied to stabilize plaques and lower cardiovascular event risks. Their therapeutic efficacy correlates with the modulation of KLF family members. For instance, simvastatin upregulates KLF2 expression in ECs, enhancing Foxp1 activity to suppress vascular inflammation and inhibit atherosclerosis progression [17]. KLF4 also mediates statin effects, as demonstrated by its role in regulating MANTIS expression. Through KLF2/KLF4 signaling, MANTIS suppresses ICAM-1-dependent monocyte adhesion to ECs, attenuating atherosclerotic development [31].

No pharmaceutical compound is entirely free from side effects, though statins play a critical role in managing atherosclerotic cardiovascular events. Common side effects include liver-related damage and mild reversible muscle symptoms such as myalgia and myositis [77]. Severe conditions like true myopathy and rhabdomyolysis are rare [78]. Most cardiovascular outcome studies targeting atherosclerosis treatment have employed statin-based combination therapies in the intervention groups. Although the efficacy is well established, the side effects and variability in individual tolerance limit the benefits for some patients. This highlights the need for exploring alternative therapeutic approaches. Therefore, the drug repositioning strategy has become an important development direction for new therapeutic approaches to atherosclerosis, serving as a beneficial complement to existing statin treatments and holding the promise of further enhancing the overall benefit rate for patients.

Metformin, a first-line therapy for type 2 diabetes, is also a classic example of drug repositioning for the treatment of atherosclerosis. Preclinical studies have shown that metformin regulates endotoxemia-induced endothelial dysfunction and inflammation through the AMPK/KLF2 axis, restoring KLF2 levels reduced by LPS and TNF-α stimulation, and inhibiting VCAM1 expression while reducing inflammatory factors like TNF-α and IL-6 [79]. In an experiment by Han and colleagues, metformin was found to enhance autophagy during atherogenesis, identifying a novel mechanism through KLF2-mediated autophagy that promotes cholesterol efflux without significantly upregulating ABCA1 [80]. Although the first metformin studies achieved positive outcomes, further experimental and clinical data will be necessary to validate the mechanisms of action and the clinical translational value of metformin in either atherosclerosis prevention or atherosclerosis treatment.

### 4.2. Potential Substances to Treat Atherosclerosis Through the KLF

Given the critical role of KLF factors in vascular health, future research is needed to explore the direct interaction between novel drugs and KLF signaling pathways to better understand their therapeutic potential in atherosclerosis.

Melatonin is a hormone secreted by the pineal gland and peripheral tissues, which has a variety of functions, and the best-known of which is to regulate the sleep–wake cycle and improve sleep quality [81]. A recent study found that knocking down PPARδ under melatonin treatment shifted VSMCs towards a synthetic phenotype and increased KLF4 expression, thereby improving plaque stability and reducing vulnerability to rupture [82]. Similarly, rapamycin improves endothelial function through activation of KLF2 when used at low doses and for short periods, which is commonly seen in drug-eluting stent applications [83]. However, at high doses or with prolonged use, rapamycin has been observed to inhibit KLF2 expression, thereby reducing the anti-inflammatory, anticoagulant, and vasodilatory properties of endothelial cells through the inhibition of the mTOR signaling pathway. This, in turn, has been hypothesized to lead to adverse cardiovascular outcomes [84]. In addition to the drugs for which there is already direct evidence of therapeutic efficacy concerning members of the KLF family, there are other potential drugs for which minocycline is used clinically, mainly as an antibiotic and an anti-inflammatory agent [60]. The field of application of minocycline is focused on infectious diseases and some immune-mediated disorders. The present study demonstrates that minocycline promotes p27KIP1 expression by inhibiting PARP-1, modulating oxidative stress, and the cell cycle. This mechanism is highly compatible with KLF4-related vaso-protective functions. Although no studies have directly examined whether Minocycline regulates p27KIP1 through the KLF4 pathway, the roles of KLF4 and Minocycline in atherosclerosis overlap. Therefore, it can be hypothesized that Minocycline activates p27KIP1 via KLF4, inhibiting VSMC proliferation and reducing plaque formation. These overlapping pathways provide the basis for a new hypothesis to investigate whether minocycline can achieve its anti-atherosclerotic effects by modulating KLF4. Besides the repositioning of medicines, the emerging therapeutic potential of KLF families towards atherosclerosis treatment urges the discovery of several novel natural or synthetic substances that can attenuate atherosclerosis by regulating KLF, which are summarized in Table 1 [26,33,85,86,87,88,89,90,91,92,93,94,95,96,97,98,99].

Beyond KLF2 and KLF4, other KLF members contribute to atherosclerosis management. Perhexiline, a KLF14 activator, inhibits macrophage inflammatory responses and enhances cholesterol efflux by increasing the expression of ABCA1 [59]. Berberine improves glucose/lipid metabolism and inflammation by promoting KLF16 nuclear translocation to activate PPARα signaling [73]. These findings underscore the potential of targeting various KLF members to mitigate atherosclerosis.

Despite promising experimental and clinical outcomes, KLF-targeted therapies face limitations. Functional redundancy among KLF family members and cell-specific actions may compromise treatment stability, while multi-target drugs (e.g., statins) risk nonspecific regulation. Additionally, precise modulation of KLF expression in diseased tissues without disrupting physiological processes remains challenging. Future studies should prioritize dose optimization and combinatorial approaches with existing KLF-targeted therapies to strengthen translational potential. Addressing these gaps will accelerate innovations in drug repurposing, gene therapy, and nanoparticle-based delivery systems (see Section 4.3), enabling rapid clinical translation of KLF-focused treatments.

### 4.3. Innovative Solutions for KLF-Targeted Therapy

#### 4.3.1. Gene Therapy

In recent years, gene therapy has demonstrated substantial potential in the treatment of various diseases, particularly cardiovascular disorders [100]. Gene therapy approaches can be broadly categorized into three main strategies: gene editing therapies aimed at achieving precise genomic modifications, gene silencing techniques using siRNA, shRNA, and miRNA, and gene replacement involving the direct delivery of plasmids and viral vectors to introduce desired genes [101].

CRISPR/Cas9 represents the latest gene editing technology that utilizes engineered RNAs to guide the Cas9 protein to specific DNA sequences, facilitating targeted genome modifications [102]. CRISPR/Cas9 is not only easier to use compared to the traditional gene-editing methods, but also, due to having an optimized gRNA design, can considerably reduce off-target effects as well [103,104]. In one experimental study, Chen and colleagues employed CRISPR/Cas9 to induce Fam172a knockout, which significantly reduced the expression of contractile markers in VSMCs, while the synthesis of the marker KLF4 was significantly increased. This resulted in the phenotypic transition of VSMCs from a contractile to a synthetic state and increased plaque area by 47.4% and heightened plaque instability, indicating that targeting Fam172a or KLF4 using gene therapy may offer a new therapeutic approach for atherosclerosis [105].

Compared to CRISPR-mediated gene editing, RNA interference allows for more reversible and controllable target silencing [106]. RNA interference, such as siRNA and shRNA, can achieve specific silencing of GPCR-like receptors (e.g., LGR4, SSTR3, GPR101, GPR116), and it has been confirmed to regulate the expression of KLF2 in vascular endothelial cells, promoting the anti-inflammatory and anti-thrombotic transcriptional programs of endothelial cells, ultimately improving endothelial function [107,108]. However, current related studies are still limited to the cellular level, necessitating further in vivo and clinical validation.

Overexpression typically involves introducing specific RNA or genes into cells or tissues to enhance their expression levels. The therapeutic effects of overexpressing certain members of the KLF family have been confirmed in animal experiments. In ox-LDL-stimulated HUVECs, overexpression of miR-148a-3p targeted KLF5 expression and inhibited the expression of inflammatory factors and functional injury [109]. Overexpression of KLF7 alleviates atherosclerosis lesions and restores metabolic abnormalities in atherosclerosis mice, while inhibiting glucose intake and glucose metabolic reprogramming in ox-LDL-induced RAW264.7 cells [63].

In summary, RNA-targeted gene therapy strategies, characterized by their diversity and specificity, enable precise regulation of target genes, showcasing a wide range of clinical application prospects [56,110,111]. Nevertheless, the clinical translation of gene therapy is still challenged by many factors, such as host immunogenicity against adeno-associated viral vectors and high research and production costs, which seriously limit patient accessibility and affordability [112]. For future programs, the emphasis should be on making gene therapy more affordable and accessible to facilitate its use in clinical applications.

#### 4.3.2. Nanotechnology

The concept of nanotechnology was first proposed by American physicist Richard Feynman in 1959, and its development began in the 1980s. At that time, the study of nanomaterials was in its nascent stages, with research gradually advancing as the nano-preparation process evolved [113]. As their unique properties became increasingly recognized, nanomaterials began to be applied across various fields. Early examples of nanomaterials, such as gold nanoparticles and quantum dots, gained widespread use in bioimaging, sensors, and drug delivery systems due to their simple structures and excellent optical properties [113,114,115]. The foundational research on these materials paved the way for the rise of nanomedicine, facilitating the initial integration of nanotechnology into the medical field.

As research into the biosafety and biocompatibility of nanomaterials progressed, scientists shifted their focus to more complex nanomaterial systems that offered greater levels of controllability [116]. These advanced nanomaterials, optimized through earlier research, have been engineered through precise design and functionalization. They not only significantly enhance drug delivery efficiency but also exhibit superior performance in targeted therapy and bioimaging [117]: (1) Nanomaterials, with their ultra-small size and large specific surface area, provide an increased number of reactive sites [118]. (2) Their unique physicochemical properties, such as excellent biocompatibility, allow them to effectively cross biological barriers and target diseased areas, greatly improving the specificity and efficiency of treatment [119]. (3) As technology continues to evolve, the design and functionalization of nanoparticles have improved, reducing off-target effects by modifying active targeting ligands, offering new potential solutions for various medical challenges [120].

The selection of appropriate nanomaterials is particularly critical in practical applications, especially in the treatment of atherosclerosis. Six types of nanomaterials are commonly used in atherosclerotic nanomedicine: micelles, liposomes, polymer nanoparticles, dendritic polymers, carbon nanotubes, and crystalline metals [121]. While more complex nanomaterials, such as nanofibers, offer structural advantages, their surface charge and rigidity can stimulate unwanted responses in the vascular endothelium, triggering inflammatory reactions and cellular stress that ultimately lead to vascular wall dysfunction [122]. In a study conducted by Yang and colleagues, exposure of multi-walled carbon nanotubes to smooth muscle cells induced the upregulation of KLF4 and KLF5, stimulating the transformation of phenotypic transformation of SMCs, and promoting plaque proliferation [123]. By contrast, nanoparticles, designed with greater precision, not only significantly enhance targeted delivery efficiency [124] but also effectively avoid overstimulation of the vascular endothelium while maintaining biocompatibility [125]. Nanoparticle-based platforms have emerged as highly promising mRNA delivery systems. In a separate study, miR-153-3p was delivered via hyaluronic acid-modified cationic liposomes (HA-CLPs) for the treatment of myocardial infarction. miR-153-3p inhibited the expression of KLF5, thereby reducing its activation of the NF-κB pathway [126]. According to Western blot analysis, the protein level of NF-κB p65 was reduced by 40–50% [127]. Ionizable lipid nanoparticles, the most extensively researched and widely used carriers for mRNA delivery, remain neutral under physiological conditions but become positively charged in acidic environments, facilitating the efficient encapsulation and release of mRNA within cells [128,129]. This ability allows for an efficient and targeted delivery of nucleic acids into cells to target KLF expression, providing a potential strategy to modulate KLF-mediated functions for disease therapy.

Nanomaterials are also employed as drug carriers in various therapeutic applications. For example, nitric oxide-releasing nanoparticles, such as GSTπ-responsive NO-releasing prodrugs, are encapsulated in redox-responsive silica nanocarriers, enabling controlled NO release in a localized high-glutathione environment characteristic of atherosclerotic plaques. This targeted delivery allows NO to activate the intracellular cGMP pathway, which in turn upregulates KLF2 expression and exerts therapeutic effects [130]. Future nanocarriers should aim to address two core issues of atherosclerosis: disordered lipid metabolism and chronic inflammation, promoting plaque degradation and, ideally, disease reversal [131,132].

**Figure 4:**
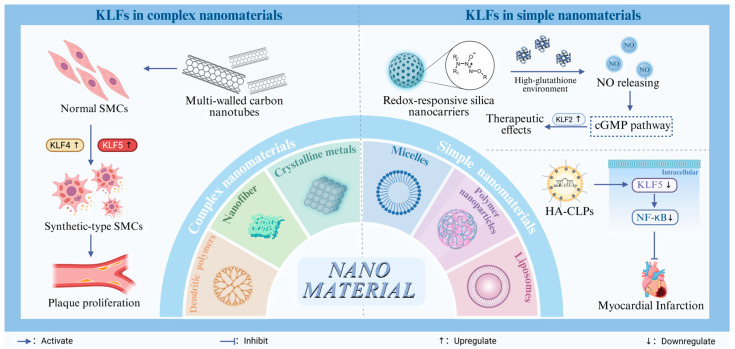
Kruppel-Like Transcription Factors (KLF) in Nanotechnology. Six types of nanomaterials are commonly used in atherosclerotic nanomedicine: micelles, liposomes, polymer nanoparticles, dendritic polymers, carbon nanotubes, and crystalline metals. Exposure of multi-walled carbon nanotubes to normal smooth muscle cells (SMCs) induced the upregulation of KLF4 and KLF5, stimulating the transformation of normal SMCs into synthetic-type SMCs and contributing to plaque proliferation, which should be avoided. Currently, there are two successful nanotechnology interventions targeting KLFs: (1) Hyaluronic acid-modified cationic liposomes (HA-CLPs) load miR-153-3p, which, after being delivered into cells, inhibits the expression of KLF5 and downregulates the NF-κB pathway. This is applied for the treatment of myocardial infarction. (2) Additionally, nitric oxide-releasing nanoparticles, such as GSTπ-responsive nitric oxide (NO)-releasing prodrugs, are encapsulated in redox-responsive silica nanocarriers, enabling controlled NO release in a localized high-glutathione environment characteristic of atherosclerotic plaques. This targeted delivery allows NO to activate the intracellular cGMP pathway, which in turn upregulates KLF2 expression and exerts therapeutic effects.

Despite the significant potential of nanomaterials in treating atherosclerosis (Figure 4), challenges persist in their clinical translation [133]. A key issue is that while treatments often show effectiveness in preclinical trials, this success is not always replicated in human clinical trials [134,135]. Additionally, individual differences may affect the efficacy of nano-delivery systems, with factors such as physiological characteristics and genetic background playing a role [136]. Developing personalized treatment protocols tailored to the unique needs of each patient remains a critical challenge for the future of nanomedicine.

## 5. Challenges and Future Directions

### 5.1. Challenges

#### 5.1.1. Redundancy and Contradiction of KLF Functions

The functional redundancy and contradictoriness of the KLF family have an impact on KLF-targeted therapy. Some members of the KLF family have overlapping functions. For example, both KLF2 and KLF4 members can protect ECs, which exerts a beneficial effect on atherosclerosis. KLF2 inhibits endothelial apoptosis through the Bcl-2 family, while KLF4 promotes endothelial cell cycle arrest and repair by regulating p21 [22]. This functional redundancy is an important dual protective mechanism of ECs.

Some members of the KLF family have both endorsing and hindering effects on the progression of atherosclerosis, which are influenced by cell- and tissue-specific, disease stage, and environmental factors. KLF6 plays a significant role in vascular endothelial protection in atherosclerosis by inhibiting inflammation, oxidative stress, and monocyte adhesion [54]. However, the downregulation of KLF6 promotes the generation of vascular protective M2 macrophages, attenuating atherosclerosis [57]. In this way, the complex functions of the KLF family should be considered during the KLF-targeted therapy to avoid adverse consequences caused by non-specific effects.

#### 5.1.2. Limited Drug Delivery Capability

The delivery efficiency of KLF-targeted therapies is another critical issue to be resolved. Traditional liposome carriers are recognized and phagocytosed by macrophages [137], making it difficult to precisely deliver drugs to atherosclerotic plaques, resulting in insufficient drug concentration at the target site and affecting therapeutic efficacy. The insufficient delivery efficiency faced by KLF-targeted therapy severely restricts its treatment outcome and clinical application.

#### 5.1.3. Inadequate Targeting of KLF Therapy

The inadequate target specificity of KLF-targeting drugs is also problematic. For example, the KLF-related drug metformin does not directly and accurately act on KLF2 to exert its anti-atherosclerosis effect [138]. This indirect mode of action leads to a wide range of actions in the body, making it difficult to focus on specific tissues to exert its adjusting function on KLF and resulting in different curative effects in different patient groups.

#### 5.1.4. Long-Term Safety Problem of KLF-Targeted Therapy

KLF-targeting therapy can induce KLF-induced regulatory mechanisms in the short term, but long-term medication may precipitate its functional disorders. For instance, KLF15 triggers the secretion of inflammatory factors such as IL-1β by holding back the TNF-α pathway, while long-term suppression of KLF15 may relieve the repression of TNF-α and result in the occurrence of inflammation [139]. Inhibiting KLF4 (using siRNA-KLF4) enhances the differentiation of vascular smooth muscle. However, prolonged intervention of KLF4 may lead to abnormal proliferation of VSMC or differentiation imbalance, thus contributing to the progression of atherosclerosis, hypertension, or vascular wall thickening [140].

### 5.2. Precision Medicine Outlook: Patient Stratification Therapy Based on KLF Expression Profiles

Regarding the redundancy of KLF, during targeted therapy, multiple KLF members with redundant functions can undergo simultaneous intervention. Screen specific inhibitors or agonists for different KLF members and use them in combination with appropriate doses to have a synergistic effect.

When applying KLF-targeted therapy, patient-stratified treatment is required to be implemented. The analysis of KLF expression levels in lesion sites using single-cell RNA sequencing and spatial transcriptomic analysis can provide a detection index for evaluating disease conditions. For example, the expressive level of KLF15 in VSMC measured by these technologies can be used in plaque stability and disease condition assessment [71]. Subsequently, precision KLF-targeted therapy based on the patient’s condition can be implemented. Notably, technologies like introducing Cleavage Under Targets & Tagmentation should be applied to reduce the cost of single-cell sequencing and ensure its scalability [141].

To enhance the efficiency of targeted delivery of KLF and tissue targeting, degrading delivery vectors is a potential solution in the future. Optimize the composition and structure of lipid carriers, increase the loading capacity of KLF-targeted drugs, modify the surface of liposomes to prevent phagocytosis by the mononuclear phagocyte system, and prolong the blood circulation time. Prepare nanogel carriers through nanotechnology to protect the drugs from premature degradation and enable slow drug release at the lesion site. Design intelligent targeting vectors for specific markers at the lesion site, such as specific receptors. Design nanocarriers to achieve accurate drug release, and external stimulus-responsive materials, such as photosensitive and magnetically sensitive materials, can be utilized to improve targeting accuracy.

For the potential safety issues of long-term use of KLF-targeted therapy, it is imperative to perform periodic and comprehensive examinations on patients, including complete blood count, routine urinalysis, assessment of hepatic and renal functions, evaluation of immunological function, determination of hormonal levels, and related tests. Then systematically contrast the indices across all phases of treatment, expeditiously discern latent safety risks, and promptly modulate the pharmacotherapy regimen in time.

## 6. Conclusions

Atherosclerosis, driven by chronic inflammation, lipid dysregulation, and vascular remodeling, remains a leading cause of global cardiovascular morbidity and mortality. This review underscores the pivotal role of KLFs in modulating key cellular processes underlying atherosclerosis, including endothelial homeostasis, SMC phenotypic switching, and macrophage polarization. Among KLF family members, KLF2 and KLF4 emerge as central players in maintaining endothelial integrity and suppressing inflammation under physiological shear stress, in contrast to the adverse effect of KLF5, while KLF6 exhibits context-dependent roles that may either exacerbate or mitigate plaque progression [22,47,54,57]. KLF14 further contributes to lipid metabolism regulation by enhancing cholesterol efflux via ABCA1, highlighting the multifaceted involvement of KLFs in atherosclerosis pathogenesis [59].

Current clinical therapies, such as statins and PCSK9 inhibitors, indirectly stimulate KLF pathways to reduce plaque burden. Emerging strategies, including gene therapy and nanotechnology, offer promising avenues for precise KLF targeting. For instance, nanoparticle-mediated delivery of miR-153-3p suppresses KLF5/NF-κB signaling [126], while nitric oxide-releasing nanocarriers activate KLF2 to restore endothelial function [130]. However, challenges persist, such as functional redundancy among KLF members, limited drug delivery efficiency, and long-term safety concerns.

Future directions emphasize precision medicine approaches, integrating multi-omics profiling to stratify patients based on KLF expression patterns. Combinatorial therapies targeting redundant KLFs and advanced nanocarriers optimized for tissue-specific delivery may enhance therapeutic efficacy. Additionally, addressing the dual roles of KLFs through spatiotemporal regulation will be critical. By bridging mechanistic insights and innovative technologies, KLF-focused interventions hold immense potential to revolutionize atherosclerosis management, ultimately reducing the global burden of cardiovascular disease.

## Figures and Tables

**Table 1:** Recently identified drugs that attenuate VC by promoting KLF.

Name	Mechanisms	Model
Resveratrol	KLF2/UCP2	Mice & HAEC & HUVECs
Ethanol extract of soy leaf	miR-92a/KLF2	Mice & HUVECs & Monocyte
Lunasin	MEK5/ERK5/KLF2	HUVECs & Monocyte
Trichostatin D	KLF2/NLRP3/ Caspase-1/IL-1β	HUVECs & Monocyte
Aloperine	P53/KLF2/LOX-1	HUVECs & Monocyte
Betulinic acid	ERK5/KLF2/eNOS	HUVECs
Flavonoids	miR-92a/KLF2	Mice & HCAEC
Phloretin	KLF2/eNOS	Mice & HUVECs
Calenduloside E	KLF2	Mice & Macrophage
Calycosin	KLF2/MLKL	Mice & Macrophage
Artesunate	KLF2/NRF2/TCF7L2	Mice & VSMCs
Formononetin	KLF4/SRA	Mice & MOVAs & HUVECs
Salidroside	KLF4/eNOS	HUVECs & Mice
Quercetin	JAK2/STAT3/KLF4	MOVAS & Mice
Antcin K	KLF4/CD36	Macrophage & ECs
Protocatechuic acid	miR-10b-KLF4-MerTK	Macrophage
Kanglexin	MEK-ERK-ELK-1/KLF4	Mice & MOVAs

Kanglexin, a new compound designed by the Harbin Medical University team, works by modulating the VSMC phenotype. KLF = Kruppel-like Transcription Factors; UCP2 = Uncoupling Protein 2; MEK = Mitogen-Activated Protein Kinase Kinase; ERK = Extracellular Signal-Regulated Kinase; NLRP3 = NOD-Like Receptor Protein 3; P53 = Tumor Protein p53; LOX-1 = Lectin-Like Oxidized LDL Receptor-1; eNOS = Endothelial Nitric Oxide Synthase; MLKL = Mixed Lineage Kinase Domain-Like Protein; TCF7L2 = Transcription Factor 7-Like 2; SRA = Scavenger Receptor A; JAK2 = Janus Kinase 2; STAT3 = Signal Transducer and Activator of Transcription 3; MerTK = MER Tyrosine Kinase; ELK-1 = ETS-Like Transcription Factor 1; HAEC = human aortic EC; HUVEC = human umbilical vein EC; HCAEC = human coronary artery endothelial cell; MOVAs = Mouse Aortic Vascular Smooth Muscle Cells.

## Data Availability

No new data were created or analyzed in this study. Data sharing is not applicable to this article.

## Abbreviations

LDL	Low-Density Lipoproteins
IL	Interleukin
TNF	Tumor Necrosis Factor
NLRP3	NOD-Like Receptor Protein 3
TGF	Tumor Growth Factor
SMCs	Smooth Muscle Cells
KLF	Kruppel-Like Transcription Factor
VSMC	Vascular Smooth Muscle Cell
HAT	Histone Acetyltransferases
CtBP	C-terminal Binding Protein
Sin3A	SIN3 Transcription Regulator Family Member A
TAD	Transactivation Domain
TRD	Transcriptional Repression Domain
EC	Endothelial Cell
eNOS	Endothelial Nitric Oxide Synthase
NO	Nitric Oxide
USS	Unidirectional Shear Stress
OSS	Oscillatory Shear Stress
CAR	Coxsackievirus-Adenovirus Receptor
AP-1	Activator Protein-1
ox-LDL	Oxidized Low-Density Lipoprotein
LPL	Lipoprotein Lipase
NRF2	Nuclear Factor Erythroid 2–Related Factor 2
TCF7L2	Transcription Factor 7-Like 2
ATF2	Activating Transcription Factor 2
lncRNA	Long Non-Coding RNA
PCD	Programmed Cell Death
Foxp1	Forkhead Box Protein P1
CAD	Coronary Artery Disease
SLC7A11	Solute Carrier Family 7 Member 11
GPX4	Glutathione Peroxidase 4
Ch25h	Cholesterol 25-Hydroxylase
LXR	Liver X Receptor
PPARγ	Peroxisome Proliferator-Activated Receptor Gamma
STAT6	Signal Transducer and Activator of Transcription 6
EndMT	Endothelial-to-Mesenchymal Transition
HVSMC	Human Vascular Smooth Muscle Cell
ABCA1	ATP-Binding Cassette Transporter A1
UCP2	Uncoupling Protein 2
P53	Tumor Protein p53
LOX-1	Lectin-Like Oxidized LDL Receptor-1
MLKL	Mixed Lineage Kinase Domain-Like Protein
SRA	Scavenger Receptor A
JAK2	Janus Kinase 2
STAT3	Signal Transducer and Activator of Transcription 3
MerTK	MER Tyrosine Kinase
MEK	Mitogen-Activated Protein Kinase Kinase
ERK	Extracellular Signal-Regulated Kinase
ELK-1	ETS-Like Transcription Factor 1
HAEC	Human Aortic Endothelial Cell
HUVEC	Human Umbilical Vein Endothelial Cell
MEF2	Myocyte Enhancer Factor 2
HCAEC	Human Coronary Artery Endothelial Cell
MOVA	Mouse Aortic Vascular Smooth Muscle Cell
HA-CLPs	Hyaluronic Acid-Modified Cationic Liposomes
